# Supplementary material for: Tuberculosis causes highly conserved metabolic changes in human patients, mycobacteria-infected mice and zebrafish larvae
Source: Sci Rep. 2020 Jul 15;10:11635. doi: 10.1038/s41598-020-68443-y (PMC7363909; doi:10.1038/s41598-020-68443-y)
Supplement: Supplementary file 1 — Supplementary information [file 41598_2020_68443_MOESM1_ESM.pdf]

Tuberculosis causes highly conserved metabolic changes in human patients, mycobacteria-infected mice and zebrafish larvae

Yi Ding, Robert Jan Raterink, Rubén Marín-Juez, Wouter J. Veneman, Koen Egbers, Susan van den Eeden, Mariëlle C. Haks, Simone A. Joosten, Tom H.M. Ottenhoff, Amy C. Harms, A. Alia, Thomas Hankemeier, Herman P. Spaink

Supplementary Figure S1

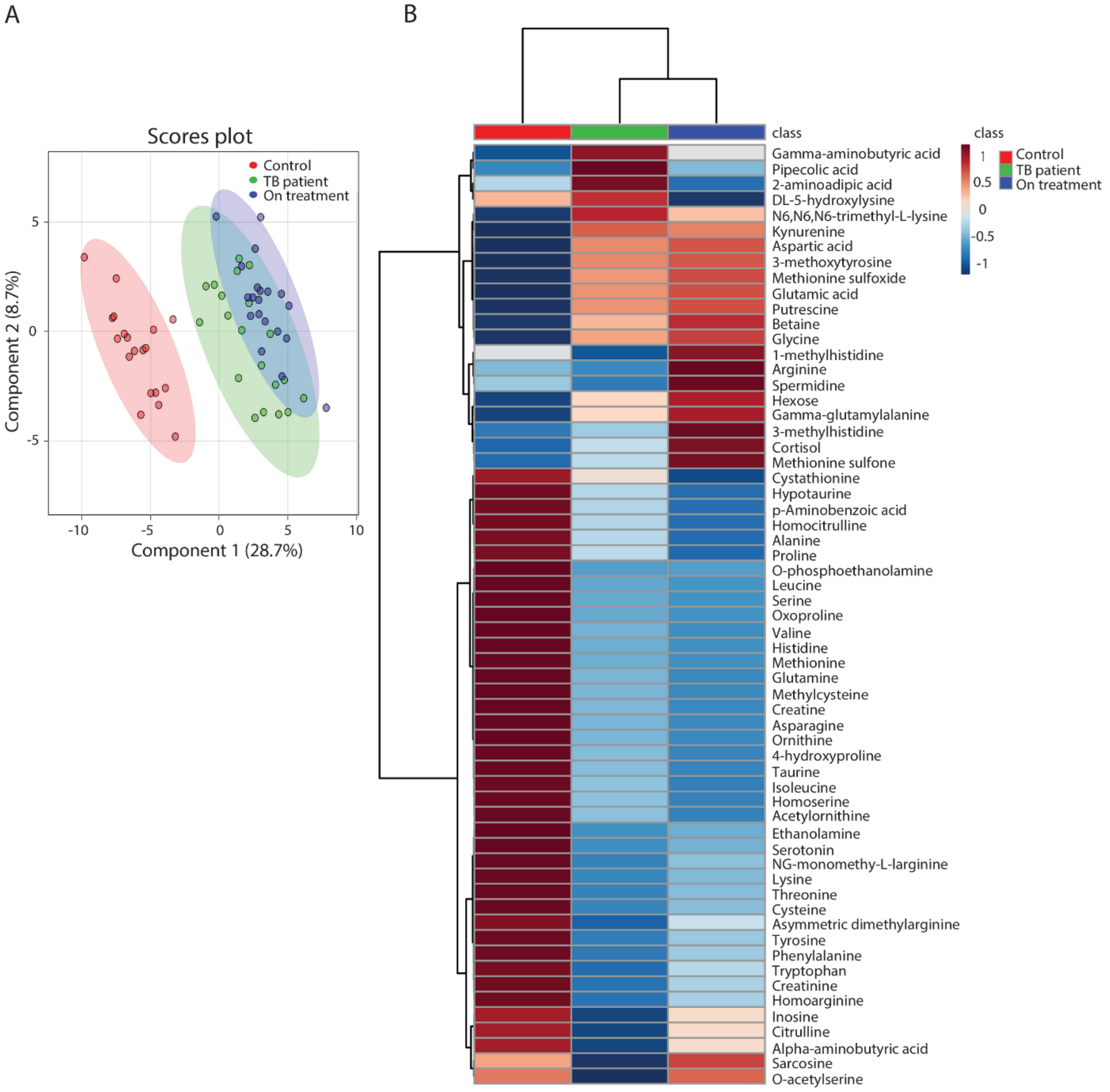

Supplementary Figure S1. Partial least squares discriminant analysis and heat map of metabolomic profiles from healthy control, TB patient, and on treatment groups. A. Analysis of blood of the healthy control group (Control), patients with active TB disease (TB patients), and the same patients treated for 6 weeks with antibiotics (On treatment), n=20. **B**. Heat map of all metabolites of blood of the healthy control group (Control), TB infected patients (TB patient) and the same patients treated for 6 weeks with antibiotics (On treatment)

Supplementary Figure S2

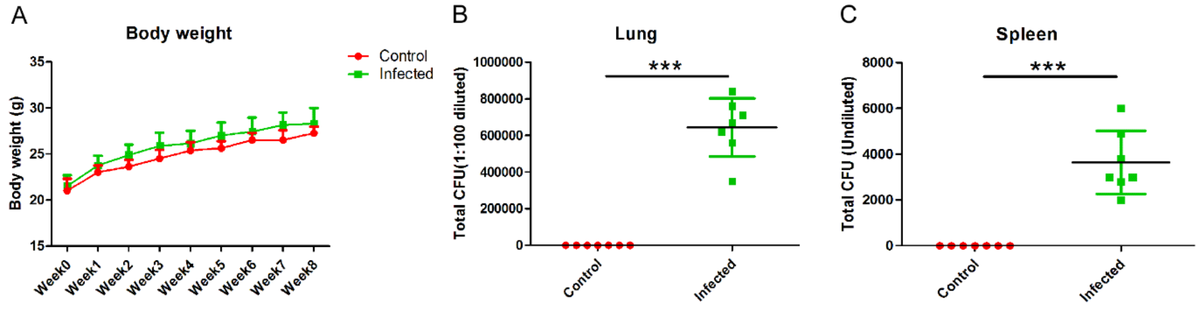

Supplementary Figure S2. Body weight and total cfu of isolated organs in mice. A. Body weight of control and infected mice from week 0 to week 8. **B.** Total cfu (1:100 diluted) of isolated lung from control and infected mice after 8 weeks of systemically infection with *Mtb*. **C.** Total cfu (Undiluted) of isolated spleen from control and infected mice after 8 weeks of systemically infection with *M.tb*. ****p*<0.001. Abbreviation: cfu, colony forming unit.

Supplementary Figure S3

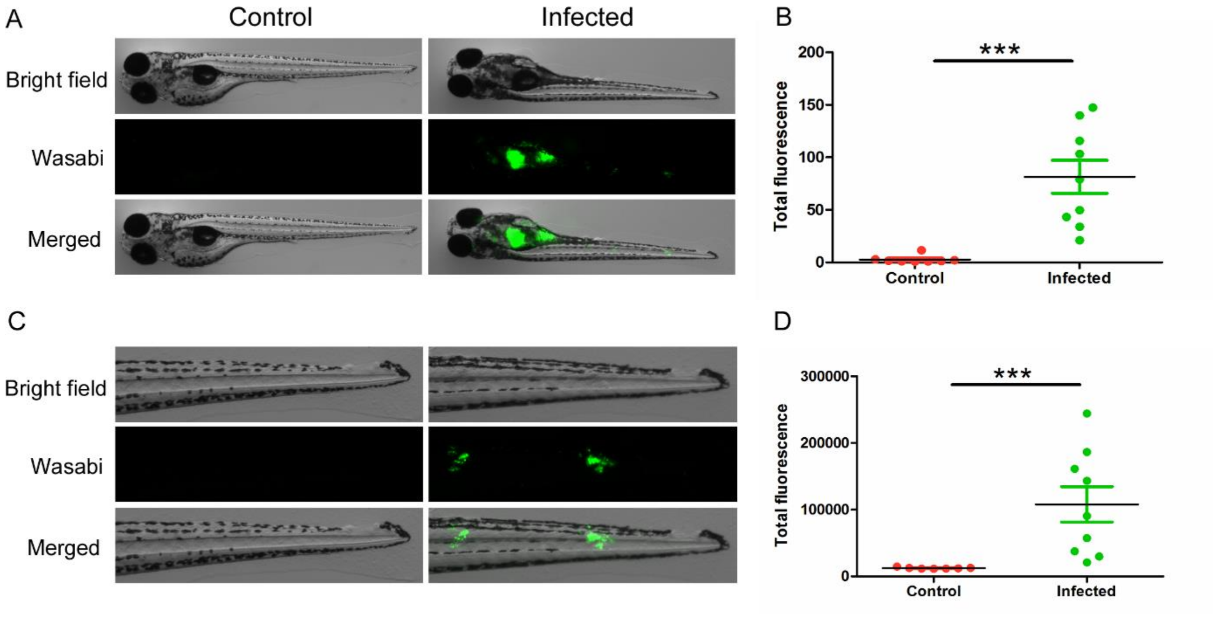

Supplementary Figure S3. Representative images and quantification of *M.m* infection in zebrafish larvae. A. The representative images of whole larva from control and infected group. **B.** Quantification of total fluorescence of wasabi from whole larva in two groups. **C.** The representative images of tail part from control and infected group. **D.** Quantification of total fluorescence of wasabi from tail part in two groups.

Supplementary Figure S4

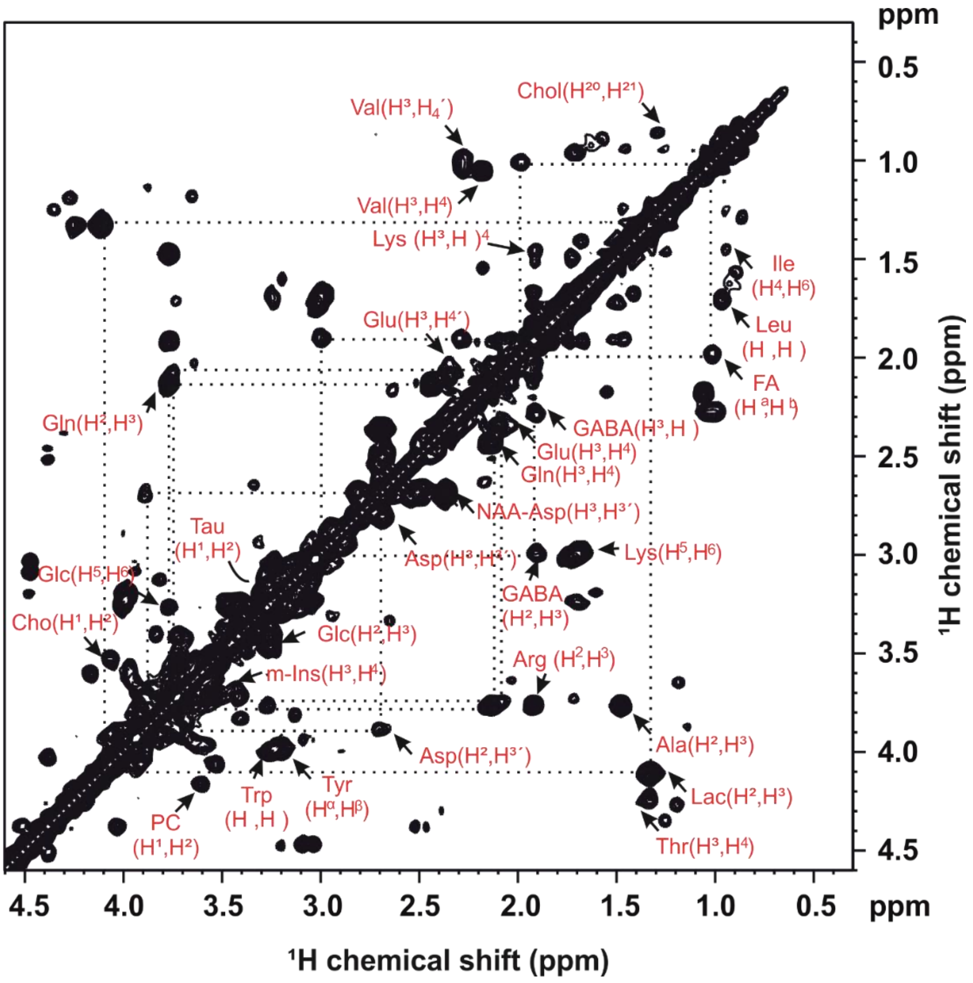

Supplementary Figure S4. Representative high resolution 2D ^{1}H-^{1}H homonuclear correlation spectrum in control zebrafish larvae. The extracted metabolites of zebrafish larvae obtained using the chemical shift correlated spectroscopic (COSY) sequence.

Supplementary Table S1.A

Gender and Age	Control n=20	TB patient n=20
Gender(male/female)	7/13	18/2
Age (years)	34.5+12.4	32.9+14.8

Supplementary Table S1.B

Sample	Label	Gender	Age
C5_1	Control	female	33
C5_3	Control	female	33
C5_7	Control	male	25
C5_10	Control	female	41

C5_13	Control	female	42
C5_14	Control	female	26
C5_15	Control	female	31
C5_18	Control	female	44
C5_20	Control	male	18
C5_21	Control	female	54
C5_24	Control	female	22
C5_25	Control	female	22
C5_27	Control	male	69
C5_28	Control	female	23
C5_29	Control	male	36
C5_30	Control	male	32
C5_32	Control	female	43
C5_33	Control	male	33
C5_34	Control	male	41
C5_38	Control	female	22
TR001	TB patient	male	22
TR002	TB patient	male	31
TR003	TB patient	male	27
TR004	TB patient	male	24
TR005	TB patient	male	44
TR006	TB patient	male	31
TR007	TB patient	male	34
TR008	TB patient	male	48
TR009	TB patient	male	41
TR010	TB patient	male	26
TR011	TB patient	male	29
TR012	TB patient	male	23
TR013	TB patient	male	21
TR014	TB patient	male	29
TR016	TB patient	female	85
TR017	TB patient	male	43
TR018	TB patient	female	32
TR019	TB patient	male	24
TR020	TB patient	male	17
TR021	TB patient	male	26

Supplementary Table S1. Gender and age information in healthy people and TB patients. **A.** Summarized gender and age information from two groups. **B.** Individual gender and age information from two groups.

Supplementary Table S2

Metabolites	HMDB identifier	Human FDR	Human Ratio
Methionine	HMDB00696	1,01E-11	0.22
Methionine sulfoxide	HMDB02005	1,47E-11	5.78
Serotonin	HMDB00259	5,35E-11	0.003
Asparagine	HMDB00168	5,35E-11	0.42
Cysteine	HMDB00574	2,47E-09	0.40
Methylcysteine	HMDB02108	4,05E-08	0.48

Hypotaurine	HMDB00965	9,09E-08	0.23
Aspartic acid	HMDB00191	4,04E-07	1.93
Glutamic acid	HMDB00148	1,04E-06	1.97
Glutamine	HMDB00641	1,21E-06	0.33
Lysine	HMDB00182	1,90E-06	0.73
O-phosphoethanolamine	HMDB00224	1,59E-05	0.0003
Threonine	HMDB00167	2,94E-05	0.66
Taurine	HMDB00251	5,84E-05	0.60
NG-Monomethy-L-arginine	HMDB29416	1,72E-04	0.57
Ethanolamine	HMDB00149	2,50E-04	0.75
Tryptophan	HMDB00929	2,55E-04	0.72
Histidine	HMDB00177	3,72E-04	0.46
Phenylalanine	HMDB00159	4,16E-04	0.79
Homoarginine	HMDB00670	7,77E-04	0.62
Alpha-aminobutyric acid	HMDB00452	9,00E-04	0.75
Putrescine	HMDB01414	1,51E-03	3.18
Citrulline	HMDB00904	2,00E-03	0.67
Homoserine	HMDB00719	2,46E-03	0.75
Serine	HMDB00187	8,84E-03	0.80
Asymmetric dimethylarginine	HMDB01539	1,16E-02	0.79
Inosine	HMDB00195	1,46E-02	0.16
Gamma-aminobutyric acid	HMDB00112	1,85E-02	1.47
p-Aminobenzoic acid	HMDB01392	2,30E-02	0.79
Creatine	HMDB00064	3,35E-02	0.67
Leucine	HMDB00687	3,50E-02	0.84

Supplementary Table S2. Ratio of metabolite quantities in blood of TB patients compared to the control group. Ratios of metabolite quantities in human blood samples. The levels of 31 metabolites are significantly altered in TB disease compared to healthy people.

Supplementary Table S3

Metabolites	HMDB identifier	Mice FDR	Mice ratio
Sarcosine	HMDB00271	4,63E-05	0.36
Ornithine	HMDB00214	1,38E-04	0.52
Proline	HMDB00162	1,92E-04	0.53
Serine	HMDB00187	3,07E-04	0.63
2-aminoadipic acid	HMDB00510	5,80E-04	0.40
Glycine	HMDB00123	5,90E-04	0.63
Gamma-glutamylalanine	HMDB06248	8,43E-04	0.47
Histidine	HMDB00177	9,87E-04	0.66
Homoserine	HMDB00719	1,21E-03	0.59
Tryptophan	HMDB00929	1,23E-03	0.48
Methionine sulfoxide	HMDB02005	1,28E-03	0.51
Ethanolamine	HMDB00149	1,31E-03	0.67
Leucine	HMDB00687	1,58E-03	0.55
Alanine	HMDB00161	1,69E-03	0.61
Citrulline	HMDB00904	2,11E-03	0.57
Phenylalanine	HMDB00159	2,62E-03	0.56
Threonine	HMDB00167	2,78E-03	0.54
Isoleucine	HMDB00172	2,94E-03	0.57

Arginine	HMDB00517	3,15E-03	0.61
Kynurenine	HMDB00183	3,47E-03	0.55
Tyrosine	HMDB00158	3,89E-03	0.52
Alpha-aminobutyric acid	HMDB00452	4,11E-03	0.65
Methyldopa	HMDB11754	4,30E-03	0.52
Gamma-aminobutyric acid	HMDB00112	4,38E-03	0.57
Valine	HMDB00883	5,51E-03	0.54
Lysine	HMDB00182	5,77E-03	0.59
Asparagine	HMDB00168	9,04E-03	0.69
Glutamic acid	HMDB00148	9,68E-03	0.64
Methionine	HMDB00696	1,79E-02	0.63
Cysteine	HMDB00192	2,36E-02	0.97
Spermidine	HMDB01257	2,63E-02	0.49

Supplementary Table S3. Ratio of metabolite quantities in blood of *Mtb*-infected mice compared to the control group. Ratios of metabolite quantities in mice blood samples. The levels of 31 metabolites are significantly altered in the infected compared to the control group.

Supplementary Table S4

Metabolites	HMDB identifier	ZF FDR	ZF ratio
Ethanolamine	HMDB00149	9,70E-07	0.41
Valine	HMDB00883	1,16E-06	0.48
Tryptophan	HMDB00929	1,38E-06	0.45
Isoleucine	HMDB00172	1,79E-06	0.42
Ornithine	HMDB00214	2,01E-06	0.58
Leucine	HMDB00687	4,97E-06	0.46
Glutamine	HMDB00641	1,10E-05	0.58
Methionine	HMDB00696	1,15E-05	0.63
Hydroxyproline	HMDB06055	2,05E-05	0.46
Phenylalanine	HMDB00159	2,16E-05	0.55
Threonine	HMDB00167	2,71E-05	0.62
Proline	HMDB00162	6,02E-05	0.67
Serine	HMDB00187	7,26E-05	0.54
Tyrosine	HMDB00158	8,11E-05	0.45
Asparagine	HMDB00168	8,31E-05	0.58
Putrescine	HMDB01414	1,08E-04	1.52
Gamma-glutamylalanine	HMDB06248	2,60E-04	0.41
Glycine	HMDB00123	3,43E-04	0.63
Arginine	HMDB00517	5,34E-04	0.65
Asymmetric dimethylarginine	HMDB01539	7,94E-04	0.54
Aspartic acid	HMDB00191	3,01E-03	0.72
Citrulline	HMDB00904	3,68E-03	0.67
Methylcysteine	HMDB02108	4,04E-03	0.48
Alanine	HMDB00161	4,35E-03	0.77
Symmetric dimethylarginine	HMDB03334	6,04E-03	0.46
Glutathione	HMDB00125	6,18E-03	1.46
3-methoxytyrosine	HMDB01434	7,16E-03	0.71
5-hydroxytryptophan	HMDB00472	8,78E-03	0.28
Beta-alanine	HMDB00056	9,06E-03	1.23
Glutathione disulfide	HMDB03337	1,22E-02	0.78

Aminoadipic acid	HMDB00510	1,37E-02	0.78
Cysteine	HMDB00192	2,25E-02	0.25
O-phosphoethanolamine	HMDB00224	2,32E-02	1.17
5-hydroxylysine	HMDB00450	3,52E-02	1.49
N6,N6,N6-trimethyl-L-lysine	HMDB01325	3,59E-02	0.62

Supplementary Table S4. Ratio of metabolite quantities in *M.marinum*-infected zebrafish larvae versus control group obtained by MS. The concentration of 35 metabolites that are significantly changed in the mycobacterial infected group compared to the control group. ZF ratio: zebrafish larvae with *M.marinum* strain E11 infection compared with control.
